# Histochemical Analysis and Ultrastructure of Trichomes and Laticifers of *Croton gratissimus* Burch. var. *gratissimus* (Euphorbiaceae)

**DOI:** 10.3390/plants12040772

**Published:** 2023-02-08

**Authors:** Danesha Naidoo, Yougasphree Naidoo, Gonasageran Naidoo, Farzad Kianersi, Yaser Hassan Dewir

**Affiliations:** 1 Department of Biological Sciences, School of Life Sciences, College of Agriculture, Engineering and Science, University of KwaZulu-Natal, Westville Campus, Private Bag X54001, Durban 4000, South Africa; 2 School of Environmental Sciences, University of Guelph, 50 Stone Road East, Guelph, ON N1G 2W1, Canada; 3 Plant Production Department, College of Food & Agriculture Sciences, King Saud University, Riyadh 11451, Saudi Arabia

**Keywords:** secretory structures, secondary metabolites, indumentum, histochemistry

## Abstract

*Croton gratissimus* (Lavender croton) possesses three distinct secretory structures. These include lepidote and glandular trichomes and non-articulated unbranched laticifers. The lepidote trichomes form a dense indumentum on the abaxial surface of the leaves and canopy the glandular trichomes. Although assumed to be non-glandular, transmission electron microscopy (TEM) indicated high metabolic activity within the stalk and radial cells. Glandular trichomes are embedded in the epidermal layer and consist of a single cell which forms a prominent stalk and dilated head. Laticifers occur on the mid-vein of leaves and are predominantly associated with vascular tissue. In the stems, laticifers are associated with the phloem and pith. Both trichome types and laticifers stained positive for alkaloids, phenolic compounds, and lipids. Positive staining for these compounds in lepidote trichomes suggests their involvement in the production and accumulation of secondary metabolites. These metabolites could provide chemical defense for the plant and potentially be useful for traditional medicine.

## 1. Introduction

Plants are an integral component of traditional medicine [1] and are used in the prevention and treatment of various ailments [2]. These therapeutic properties are due to biologically active compounds such as alkaloids, tannins, saponins, flavonoids, phenols, glycosides, terpenoids, anthocyanins, and coumarins produced by plants [3]. Plant secretory structures are involved in the production of natural bioactive compounds [4,5]. Almost all plants possess tissues or organs which primarily produce and store secondary metabolites [4,5,6]. These secretory structures comprise of either single or multiple cells that vary in structure, topography, and substance secreted [5]. Based on their location, they are classified as external or internal structures [5,6,7]. Trichomes/papillae and glands, nectaries, osmophors, hydathodes, and colleters are examples of external secretory structures. Secretory cells/idioblasts, cavities, ducts, and laticifers are classed as internal secretory structures [7,8].

Within the Euphorbiaceae, trichomes and laticifers are important features used in taxonomy [9]. Trichomes are minute appendages that arise from the epidermal cells of aerial plant parts [10]. These structures are categorized into non-glandular and glandular, based on their secretory abilities [11,12,13]. Non-glandular trichomes are presumed to be non-secretory whilst glandular trichomes produce copious amounts of secondary metabolites [6,11,12,13,14,15]. Laticifers are internal secretory systems that comprise specialized single or multiple cells [16,17,18]. They are categorized into two major types: non-articulated which arise from a single cell and articulated that develop from multiple cells [16,19]. These structures are responsible for synthesizing and accumulating latex [18,20] which is released upon damage of the laticiferous tissue [20,21]. Latex contains various secondary metabolites, including alkaloids, terpenoids, and phenolic compounds [19,20,21]. Latex-bearing *Croton* species are used traditionally to treat wounds, gastric ulcers, rheumatism, and intestinal inflammation [22].

*Croton gratissimus* Burch. var. *gratissimus* (syn. *C. zambesicus* Müll. Arg.; *C. microbotryus* Pax., *C. amabilis* Müell. Arg.), commonly known as lavender *Croton* or lavender fever berry, belongs to the family Euphorbiaceae [23,24,25]. It is a semi-deciduous shrub or tree with widespread distribution in tropical, central, and sub-Saharan Africa [23,24,26]. This species has been used extensively in traditional medicine to treat various ailments such as fever, uterine disorder, dysentery, pleurisy, convulsions, chest complaints, bleeding gums, and malaria [24,26,27,28]. Many phytochemical investigations have been conducted on *C. gratissimus* to validate its therapeutic properties. However, research on the location of the structures involved in metabolite production is lacking. In this study, we identify and describe the micromorphology of trichomes and laticifers from the leaves and stems of *C. gratissimus* and determine the phytochemical compounds produced.

## 2. Materials and Methods

### 2.1. Plant Collection and Sampling

The leaves and stems of naturally growing *Croton gratissimus* Burch. var. *gratissimus* was collected from the University of KwaZulu-Natal, Westville Campus (29°49′08.1″ S 30°56′38.9″ E), and a voucher specimen was deposited in the Ward Herbarium (*Croton* 01—Accession No. 18224). The leaves were classified into three developmental stages (N = 20): Emergent (<30 mm), young (30–60 mm), and mature (>60 mm).

### 2.2. Stereomicroscopy

To obtain a general overview, the stem, abaxial, and adaxial surfaces of fresh whole leaves (≈6 for each developmental stage) were viewed using a Nikon AZ100 (Tokyo, Japan) stereomicroscope equipped with a Nikon DS-Fi3 camera. Images were captured at different magnifications using the NIS-Elements D 4.00 imaging software.

### 2.3. Scanning Electron Microscopy (SEM)

A Zeiss Leo 1450 SEM was used to examine the micromorphology of the trichomes and laticifers present on and in the leaves and stems. Chemical fixation and freeze-drying were employed to prepare the material for viewing.

#### 2.3.1. Chemical Fixation

Fresh leaves from each developmental stage and stems (≈10 each) were rinsed in a 1% sodium hypochlorite solution to clean surfaces, and the abaxial surface of some leaves was stripped with cellophane tape to view the underlying trichomes. Thereafter, leaf and stems were cut into smaller sections (≈3 mm × 4 mm) and fixed in 2.5% glutaraldehyde for 24 h at 4 °C before being subjected to three phosphate buffer (0.1 M with a 7.2 pH) washes (5 min each). This was followed by post-fixation in 0.5% osmium tetroxide for 4 h at room temperature. Thereafter, the material underwent three phosphate buffer rinses (5 min each) and was then dehydrated in a graded series of ethanol, 30%, 50%, 70% (each twice for 5 min), and 100% (twice for 10 min). Following dehydration, samples were dried with a Quorum K850 Critical Point Dryer. Chemically fixed leaf and stem fragments were then mounted onto aluminum stubs which were secured with carbon conductive tape and sputter-coated with gold in a Quorum Q150 RES gold Sputter Coater. Samples were viewed using a Zeiss Leo 1450 SEM (Oberkochen, Germany) at a working distance of 18 mm. Images were captured using the SmartSEM imaging software.

#### 2.3.2. Freeze-Drying

Stem and leaf fragments (from each developmental stage) were rinsed in 1% sodium hypochlorite and quenched in liquid nitrogen. The sections were then fractured on metal discs submerged in liquid nitrogen before being freeze-dried in an Edwards EPTD3 Freeze-Dryer at −60 °C (vacuum pressure 10^−2^ Torr for 96 h). Freeze-dried samples were then mounted onto aluminum stubs with carbon cement and coated with gold in a Quorum Q150 RES gold Sputter Coater. Viewing and imaging of the samples was achieved using a Zeiss Leo 1450 SEM at 15 mm working distance and SmartSEM imaging software, respectively.

### 2.4. Sample Preparation for Light and Transmission Electron Microscopy (TEM)

Approximately 12 fresh leaves (from each developmental stage) and stem fragments (2 mm^2^) were chemically fixed as per SEM chemical fixation protocol. However, samples were dehydrated in a graded series of acetone solutions ranging from 30%, 50%, 75%, and 100%, with two 10 min changes for each. An additional dehydration step was carried out by washing the material twice with propylene oxide for 10 min each. Following dehydration, the samples were gradually infiltrated with Spurr’s resin [29] (propylene oxide; 1:3, 1:1, 3:1) before whole resin infiltration (100%) for 24 h. The samples were then orientated in silicone molds which were filled with whole resin. The resin blocks were then allowed to polymerize for 8 h at 70 °C before being sectioned with glass knives. Semi-thin sections (1 µm) from the resin blocks were obtained using a Leica EM UC7 Ultra Microtome. The sections were fixed onto glass slides and stained with Toluidine blue O. Prepared slides were viewed using a Nikon Eclipse 80*i* compound and fluorescent microscope equipped with a Nikon DS-Fi1camera. Images were captured using the NIS-Elements D 4.00 software. Sections (100–130 nm) were cut using a Leica EM UC7 Ultra Microtome post-stained. The copper grids (5 grids per sample) were placed onto drops of uranyl acetate and allowed to stain for 10 min before being rinsed with fresh distilled water. The grids were then placed onto drops of lead citrate enclosed in a petri dish with sodium hydroxide pellets and stained for a further 10 min. Thereafter, grids were rinsed with distilled water and dried on filter paper. Stained sections were viewed using a JEOL 1010 TEM (Japan). Images were captured on the iTEM software.

### 2.5. Fluorescence Microscopy

Transverse sections (80–100 µm) of fresh leaves and stems were cut using an Oxford^®^ Vibratome Sectioning System. Sections were stained, mounted onto glass slides with distilled water, and viewed using a Nikon Eclipse 80i compound and fluorescent microscope equipped with a Nikon Super High-Pressure Mercury Lamp and a Nikon DS-Fi1camera. Images were captured on the NIS-Elements D 4.00 software.

#### 2.5.1. Acridine Orange

Sections were stained with 0.01% aqueous acridine orange for 20 min before being rinsed with distilled water for the detection of acidic compounds such as nucleic acids and lignin. Stained sections were viewed at a wavelength of 488 nm under blue light. Lignified cell walls emitted a yellow–green fluorescence whilst non-lignified cells fluoresced red [5].

#### 2.5.2. Auto-Fluorescence

Unstained sections were viewed with ultraviolet (UV) light with an excitation wavelength of 330 nm. Two types of fluorescence are generated at UV excitation wavelengths between 340–360 nm. Phenolic compounds emit a blue fluorescence. Chlorophyll emits a red fluorescence which indicates the presence of chloroplasts [30,31].

### 2.6. Histochemistry

Fresh leaves and stems were sectioned transversely (80–100 µm) using a Vibratome. Sections were stained, mounted onto glass slides, and viewed using a Nikon Eclipse 80i compound and fluorescent microscope equipped with a Nikon DS-Fi1camera. Images were captured on NIS-Elements D 4.00 software. The following compounds were identified and localized using appropriate histochemical stains.

#### 2.6.1. Alkaloids

Sections were stained with Wagner’s reagent for 20 min before being rinsed with distilled water. A brown/orange color indicated the presence of alkaloids [5,32].

#### 2.6.2. Lipids

##### Sudan III

Sections were placed in a saturated solution of Sudan III for 15 min and then rinsed with 70% ethanol to remove excess stain. Lipids stained red/orange [33,34].

##### Nile blue A

Sections were immersed in Nile blue A and stained for 5 min at 60 °C. Thereafter, the sections were washed twice in 1% acetic acid at 60 °C, followed by rinsing in distilled water. Acidic lipids stained blue whilst neutral lipids stained pink [5].

#### 2.6.3. Phenolic Compounds

Sections were placed in 10% ferric chloride and allowed to stain for 30 min. Thereafter, sections were washed with distilled water to remove excess stain. Brown/black precipitate was a positive indicator for the presence of phenolic compounds [5].

#### 2.6.4. Lignin

Sections were immersed in 10% phloroglucinol for 15 min and then mounted in 25% hydrochloric acid and viewed microscopically. Lignin was stained pink/red [5].

#### 2.6.5. Polysaccharides (Pectin and Mucilage)

Sections were flooded with aqueous Ruthenium Red solution (1:5000) for 10 min. Acidic polysaccharides (pectinaceous substances and mucilage) stained red/pink [35].

#### 2.6.6. Carboxylated Polysaccharides, Polyuronides, Macromolecules with Free Phosphate Groups, and Polyphenols

Sections were placed in 0.05% Toluidine blue O (metachromatic stain) for 1 min before being rinsed with distilled water. Carboxylated polysaccharides and polyuronides stained pinkish purple, Macromolecules with free phosphate groups stained purple or green–blue and polyphenols (lignins) stained green/bright blue [36,37].

## 3. Results and Discussion

### 3.1. Surface Overview

**Figure 1:**
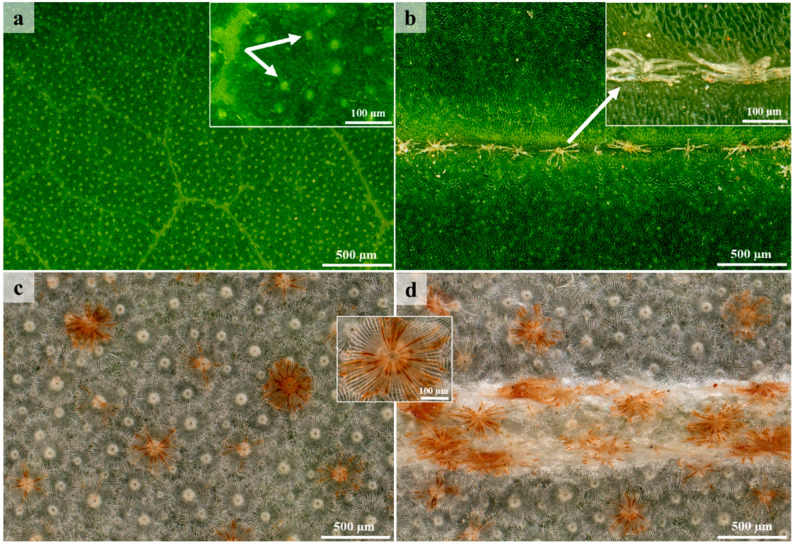

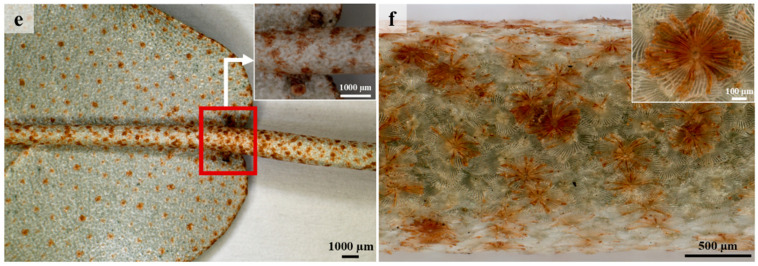
Stereomicrographs of stem and leaf surfaces. (**a**) Glabrous lamina showing translucent dots on adaxial surface. (**b**) Stellate trichome along sunken mid-vein on the adaxial surface. (**c**) Lamina of abaxial surface densely covered with lepidote trichomes. (**d**) Mid-vein on abaxial surface covered with lepidote trichomes. (**e**) Extrafloral nectaries present on the mid-vein at the base of the leaf. (**f**) Dense indumentum of lepidote trichomes on stem.

**Figure 2:**
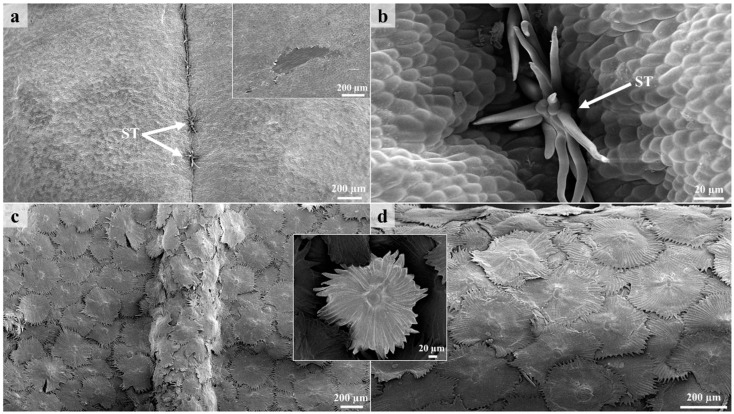
Scanning electron micrographs of leaves and stems. (**a**) Adaxial surface showing stellate trichomes along the mid-vein of leaf. (**b**) Stellate trichome emerging from middle furrow (mid-vein) on adaxial surface. (**c**) Dense indumentum formed by lepidote trichomes on the lamina and mid-vein on the abaxial surface. (**d**) Lepidote trichomes fully covering stem. ST—Stellate trichome.

Stereomicrographs provided a general overview of the stems and leaf surfaces at the different developmental stages. At low magnifications, the adaxial surface of the leaves was glabrous and shiny, indicating the presence of a cuticle layer above the epidermis (Figure 1a,b). The cuticle layer plays an important role in preventing water loss from plant surfaces [38]. At higher magnifications, the lack of pubescence on the lamina of the adaxial surface was clearly visible (Figure 1a and Figure 2a). Stereomicrographs of the adaxial surface revealed the presence of translucent dots, which were not observed under SEM (Figure 1a). However, these dots were not studied further. Stellate trichomes were present on the adaxial surface of all developmental stages, along the sunken mid-vein of the leaves (Figure 1b and Figure 2a,b). The stems and abaxial surfaces of leaves were densely covered with lepidote trichomes, resulting in a silvery appearance (Figure 1c–f). Many species in *Croton* possess a characteristic silver indumentum with copper specks formed by scale-like trichomes on the abaxial surface [39,40]. This dense indumentum protects developing leaves from desiccation as leaves are folded inwards, exposing the abaxial surface [41]. Lepidote trichomes did not decrease with leaf maturity. Some of the lepidote trichomes were orange/brown and were visible as rust specks on the stems and abaxial surfaces of leaves (Figure 1c–f). The lepidote trichomes formed a dense indumentum on the stems and abaxial surface of leaves (Figure 1c–f) and covered the underlying glandular trichomes. The latter were only visible when the lepidote trichomes were removed. The dense indumentum may provide protection for the leaf and for the smaller, glandular trichomes [42]. The lepidote and glandular trichomes were abundant at all developmental stages. The adaxial surfaces of leaves also possessed non-glandular stellate trichomes along the sunken mid-vein (Figure 1b and Figure 2a,b). The trichomes also formed an indumentum on the petioles (Figure 1e). Overlapping of lepidote trichomes on the abaxial surface of leaves and stems was observed at higher magnification (Figure 2c,d). Extrafloral nectaries were present on the mid-vein at the base of the leaf on the abaxial surface (Figure 1e). Nectaries are common in *Croton* and provide rewards to insects that defend the plant against herbivores [41]. Nectaries are also covered with lepidote trichomes. The lepidote and glandular trichomes present on the stems and abaxial surfaces of leaves are two of the seven types of trichomes that have been identified and described in *Croton* [43].

### 3.2. Lepidote Trichomes

Lepidote trichomes are scale-like hairs that are common in *Croton* species and resemble the appressed-stellate trichomes. However, the radial cells of lepidote hairs are fused, while those of stellate, are not, resulting in their shield-like appearance [43]. Lepidote trichomes function to increase water uptake from the atmosphere as the shield-like structure provides a larger surface area for absorption [44]. In addition, the dense indumentum formed by lepidote trichomes may also function to protect the plant from herbivores, pathogens, excessive water loss, increased temperatures, and UV radiation [11,42,45].

**Figure 3:**
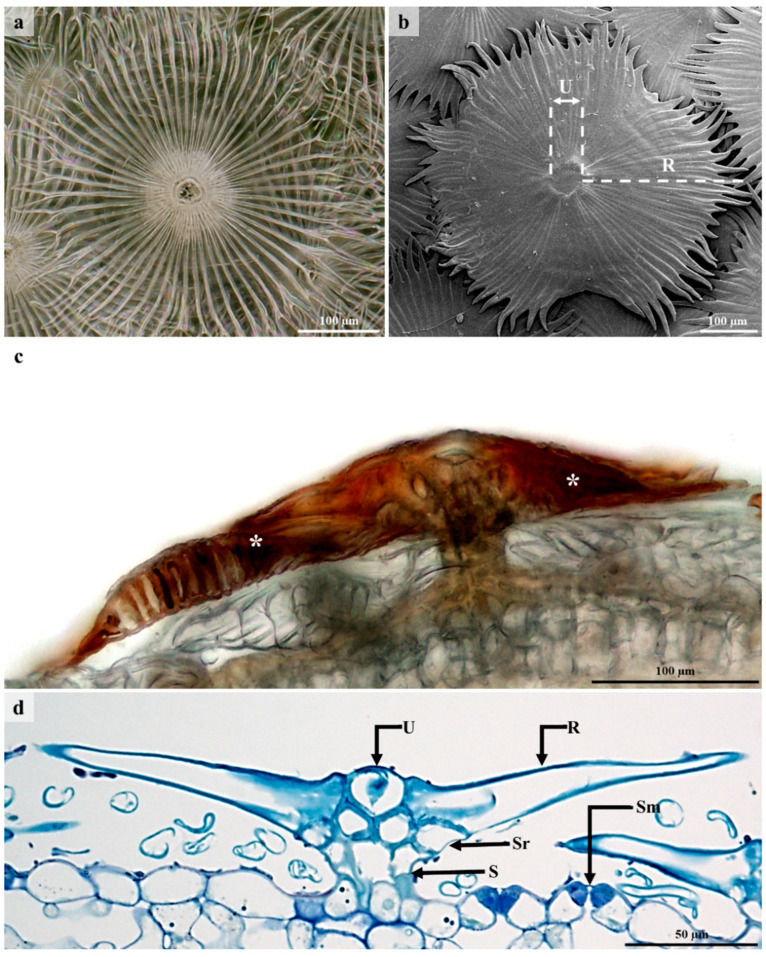
Morphology of lepidote trichomes. (**a**) Stereomicrograph of lepidote trichome. (**b**) SEM of lepidote trichome showing umbo/central cell and numerous webbed radial cells. (**c**) Lepidote trichome with dark brown accumulated secretory substance. (**d**) Light micrograph of lepidote trichome showing stalk cells, subradial cells, radial cells, and umbo/central cell. U—Umbo, R—Radii/Radial cell, Sr—Subradial cell, S—Stalk, Sm—Stoma.

**Figure 4:**
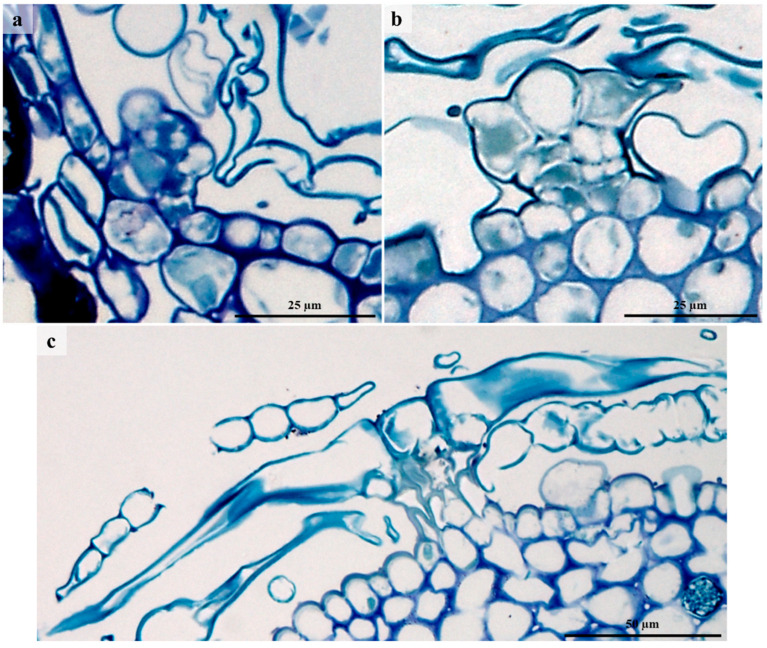
Development of lepidote trichomes. (**a**) Emergence of protodermal cells giving rise to lepidote trichome through periclinal and anticlinal divisions. Periclinal divisions initiate the development of the stalk and the anticlinal divisions of the radial cells surrounding the central cell. (**b**) Trichome emergence. (**c**) Fully developed lepidote trichome with prominent stalk, developed subradial cells, extended radial cells, and distinct central cells. U—Umbo, R—Radii/Radial cell, Sr—Subradial cell, S—Stalk.

The lepidote trichomes develop through a series of anticlinal and periclinal divisions [44]. These divisions produce a multicelled stalk whilst the stretching of the lateral cells results in the radial cells. The resultant structure comprises a multiseriate, multicellular stalk, a multicellular subradial disc, numerous radial cells, and a unicellular umbo/central cell (Figure 3 and Figure 4), similar to the lepidote trichomes in *Croton erythroxyloides* [44]. The radial cells of the lepidote trichomes are connected by their cell walls (Figure 3b) ranging between 80–100% fusion, giving it a webbed appearance. Webster et al. [43] developed an arbitrary scale to distinguish between the various types of lepidote trichomes. This scale included lepidote trichomes transitioning from stellate types with little webbing to hairs with radii that are completely fused. Fully developed and developing lepidote trichomes were present on leaves and stems because of the asynchronisation and early development of these emergences [44]. Developing trichomes were canopied by the mature lepidote trichomes (Figure 4a–c).

### 3.3. Glandular Trichomes

**Figure 5:**
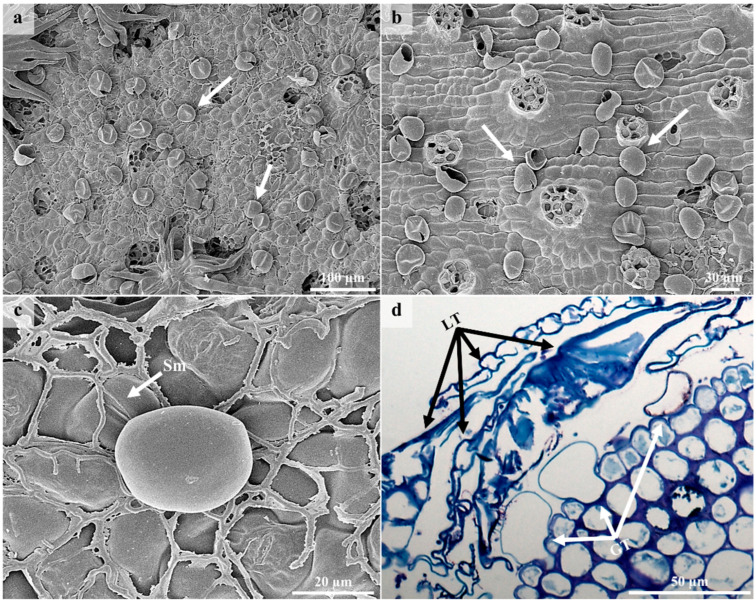
SEM and LM micrographs showing glandular trichomes on the leaves and stems. (**a**) Glandular trichomes on abaxial surface of leaves beneath lepidote trichomes. (**b**) Stem showing glandular trichomes after removing lepidote trichomes. (**c**) High magnification of single glandular trichome on abaxial surface of leaf. (**d**) Light micrograph showing unicellular glandular trichomes of different forms canopied by several layers of lepidote trichomes. Sm—Stoma, LT—Lepidote Trichome, GT—Glandular Trichome.

According to literature [11,12,13,14], glandular trichomes are involved in the production, secretion, and accumulation of various secondary metabolites. Glandular trichomes were observed on the abaxial surfaces of leaves and stems (Figure 5). Other studies also reported the presence of secretory trichomes on the abaxial surfaces of leaves in other *Croton* species [41]. In our investigation, secretory structures also occurred on the extrafloral nectaries. According to Webster et al. [43], glandular trichomes exist in a limited number of *Croton* species and may occur on either one or both leaf surfaces. These authors described glandular trichomes as “small embedded epidermal glands” and suggested that they contain terpenes, which are responsible for the aroma when the leaves are crushed. The glandular trichomes were canopied under layers of lepidote trichomes (Figure 5d). Light micrographs indicate that they comprise a single cell and are embedded in the epidermal layer (Figure 5d). These unicellular glandular trichomes formed a prominent stalk and dilated head (Figure 5d), which is consistent with the secretory trichomes of *Croton* species [41]. The glandular trichomes existed in various forms because of space limitations posed by the dense lepidote trichomes. Light microscopy and SEM indicated paracytic stomata on the abaxial surface of leaves (Figure 3d and Figure 5c). These stomata are a common character in Euphorbiaceae. Paracytic stomata are considered primitive, whilst anomocytic, diacytic, anisocytic, and parallelocytic are more advanced [46]. A study by de Sá-Haiad et al. [47] revealed that paracytic stomata are predominant in *Croton* species.

### 3.4. Laticifers

**Figure 6:**
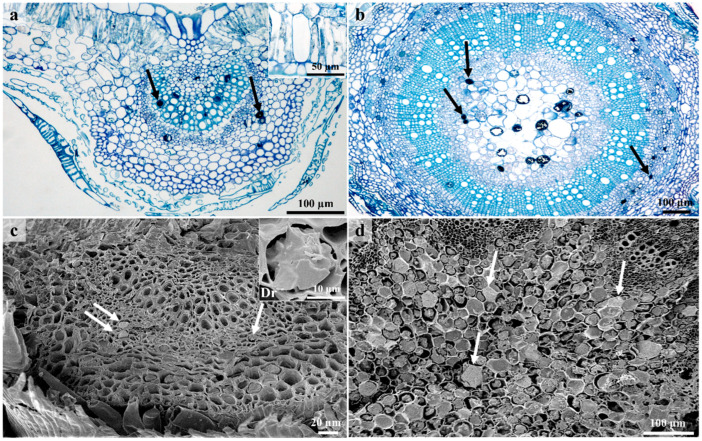
Laticifer distribution in leaves and stems. (**a**) Transverse section of leaf stained with Toluidine blue O showing distribution of laticifers predominantly in the vascular tissue. Note the idioblasts at the adaxial side of the leaf. (**b**) Transverse section of the stem stained with Toluidine-Blue showing laticifers in the phloem and pith. (**c**) Scanning electron micrograph of coagulated latex within laticifer cells (associated with phloem). Druse are also present in the leaf section. (**d**) Transverse section through stem showing latex containing laticifers in pith. Id—Idioblast, Dr—Druse, black arrows—Laticiferous cells, white arrows – latex within laticiferous cells.

**Figure 7:**
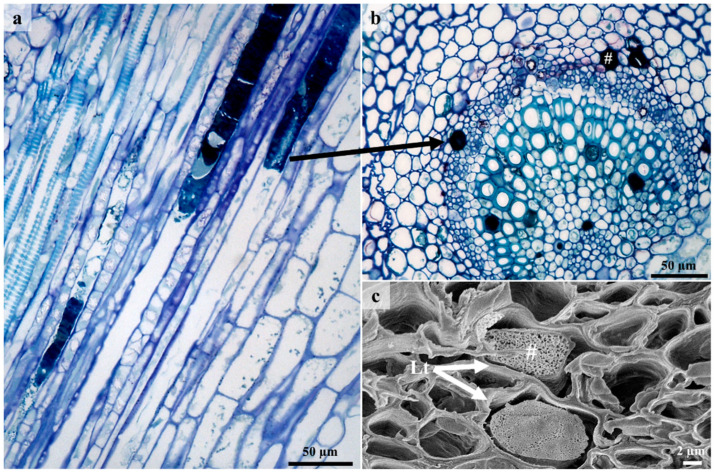
Laticifer cells showing secretory contents. (**a**) Longitudinal section of leaf showing latex within non-articulated laticifers. (**b**) Light micrograph of transverse section showing laticifer cells with latex contents. (**c**) Freeze—fracture through laticifer cells containing coagulated latex. Arrow – laticiferous cells containing latex, Lt—Laticifer, #—Latex.

A single laticifer type was observed on stems and leaves (Figure 6 and Figure 7). Non-articulated unbranched laticifers present in *C. gratissimus* were predominantly associated with the vascular tissue in the leaves and the phloem and pith in the stems (Figure 6 and Figure 7). Within the Euphorbiaceae, latex and laticifer distribution are characters used to classify the family [48]. The laticifers present in the mid-vein were predominantly associated with the vascular tissue and occasionally with parenchyma (Figure 6a,c). In the stems, the laticifers were predominant in the phloem and pith (Figure 6b,d). Laticifers are typically associated with the vascular tissues, more specifically the phloem, but may also occur in the stem pith, cortex, and foliar mesophyll [8,20,21]. However, in this study, laticifers were not observed in the foliar mesophyll. Both non-articulated (branched and unbranched) and articulated laticifers have been reported in Euphorbiaceae [19,49]. However, non-articulated laticifers are more common and widespread compared to the articulated type [49]. In our study, laticifers appeared non-articulated and unbranched and composed of a single row of cells (Figure 7a). According to Lange [20], non-articulated laticifers are cells that are secretory structures that develop from a single cell through apical intrusive growth [50]. The cell divides ceonocytically, resulting in an elongated, multinucleated structure [20,51]. Longitudinal and transverse monitor sections stained with Toluidine blue O revealed latex within laticifer cells (dark stained contents) (Figure 7a,b). Fresh latex from the leaves and stems of *C. gratissimus* was difficult to identify as the exudate was a clear, watery sap. The protoplast of laticiferous cells is the latex, which contains the metabolites and is housed within a larger central vacuole [21,52]. These compounds may function to protect the plant against herbivores and pathogens [52]. SEM of freeze-fractured material also indicated latex within laticifer cells (Figure 7c). Coagulation of the latex within the cells was probably due to a decrease of turgor within cells during tissue preparation. Generally, the pressure of latex within laticifer cells is high. When there is a sudden drop in pressure, the surrounding turgid cells compress the laticiferous cell, releasing the latex [53] which polymerizes when exposed to air [52]. This coagulation of latex seals plant wounds [54].

Light and SEM micrographs also indicated druse (Figure 6c) and prismatic forms of calcium oxalate crystals in the leaves. These calcium oxalate crystals [55,56] are housed within vacuoles of specialized cells known as crystal idioblasts [57]. Within a crystal idioblast, there is great variation in the number, shape, and size of the crystals [55,58]. However, common shapes include the druse, styloid, raphide, prism, and crystal sand [55,58]. These crystals have been used as taxonomic tools due to the specificity of the shape and location within a taxon [55,58]. Calcium oxalate is present in many forms in various genera of Euphorbiaceae [59]. Calcium oxalate crystals appear to have various functions, including removing excess calcium and oxalate to maintain ionic balance and prevent toxicity, providing tissue support, and protection against foraging herbivores [55,56,60].

### 3.5. Histochemistry and Fluorescence Microscopy

**Figure 8:**
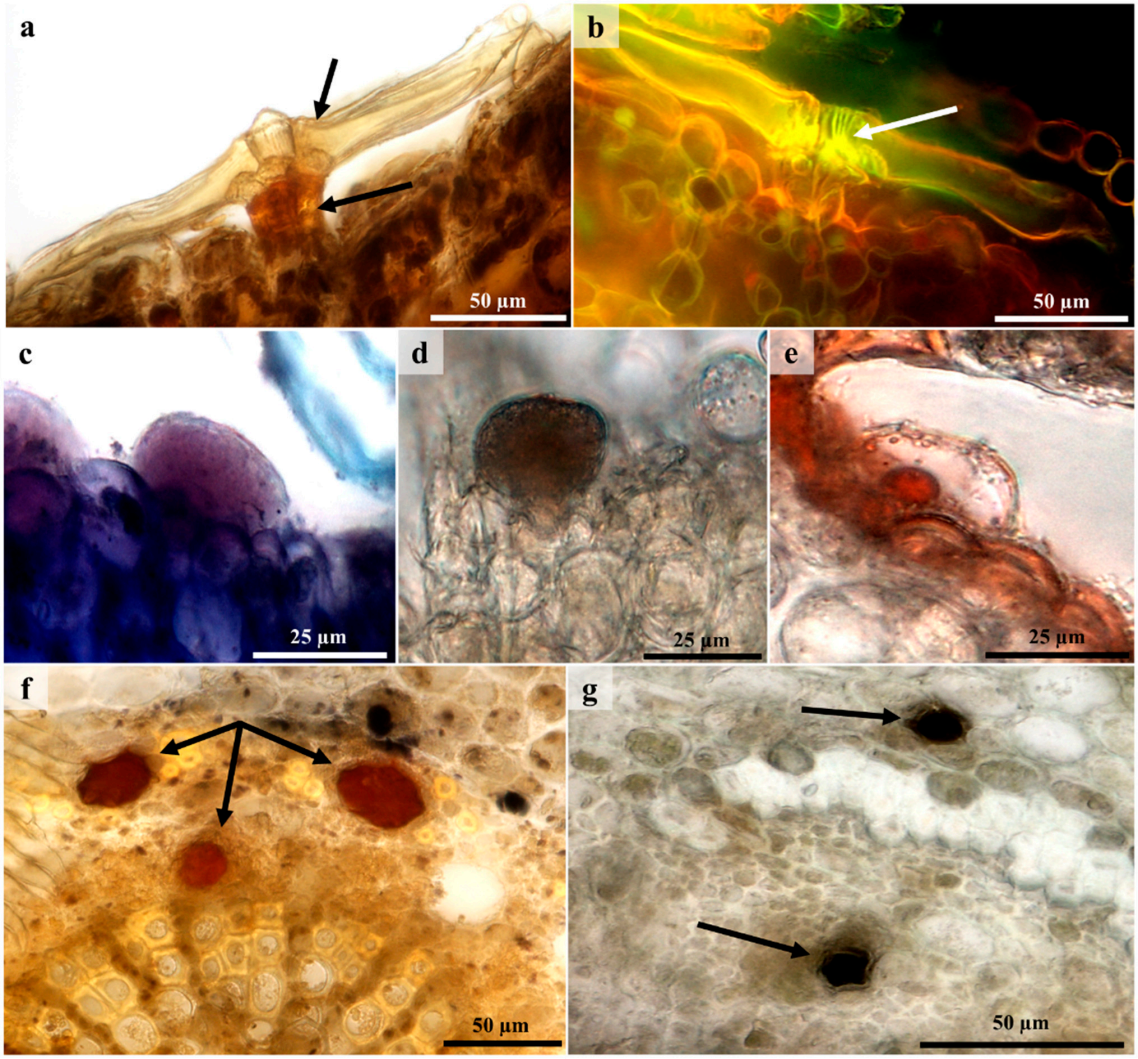
Histochemical and fluorescence micrographs showing chemical compounds of lepidote and glandular trichomes, and laticifers. (**a**) Orange/brown coloration of stalk (intense) suggests a positive indication for the presence of alkaloids with Wagner’s reagent. (**b**) Yellow fluorescence with acridine orange revealed lignified central cells in lepidote trichomes. (**c**) Pink coloration inside glandular trichome indicated neutral lipids with Nile Blue. (**d**) Glandular trichomes tested positive for phenolic compounds with ferric chloride (indicated by brown/black precipitate). (**e**) Lipid droplet of glandular trichome stained red/orange with Sudan III. (**f**) Orange coloration of laticiferous cells is a positive indication for alkaloids with Wagner’s reagent. (**g**) Positive indication (dark brown to black) for phenolic compounds in laticiferous cells stained with ferric chloride. Arrows – Positive reactions for stains.

Histochemical and fluorescence analyses of the lepidote and glandular trichomes and laticifer cells revealed the presence of hydrophilic and lipophilic substances. The presence of these secondary metabolites indicates that lepidote and glandular trichomes and laticifer cells may be responsible for the production of biologically active compounds that are used in traditional medicine [22]. Although lepidote trichomes are generally regarded as non-glandular [61,62,63] and non-secretory [11], they tested positive for various compounds (Figure 8 and Table 1).

In lepidote trichomes, subradial and central cells appeared lignified after staining with Toluidine blue O and phloroglucinol. A bright yellow fluorescence emitted by these cells after staining with acridine orange also indicated the presence of lignified cells (Figure 8b). A study by Vitarelli [44] identified lignified central cell walls in *Croton erythroxyloides*. Cells that are lignified or cutinised typically act like endodermal cells that prevent the apoplastic flow of water or the backflow of secreted substances [16,42]. The cell walls of the subradial, central, and radial cells of lepidote trichomes contained pectinaceous substances as they were stained pink with Ruthenium Red. The pectin provides support and strengthens these trichomes. Pectin may also aid in plant defense, as it induces phytoalexin accumulation which possesses antimicrobial properties [64]. Both trichome types and laticifers possessed alkaloids, phenolic compounds, and lipids. Lepidote trichomes (stalk) (Figure 8a), glandular trichomes, and laticifers (Figure 8f) stained orange/brown with Wagner’s reagent, indicating the presence of alkaloids. *Croton* species have been reported to contain an abundance of active alkaloids [22]. Alkaloids are common among angiosperms and are considered to be the most active, diverse, and therapeutic secretory compounds [65,66]. Their main function is to provide chemical defense against herbivores and pathogenic microorganisms [65,66,67]. In addition, plants containing alkaloids are used to treat various ailments due to their medicinal and pharmacological properties [66,67,68].

Positive reactions for phenolic compounds were observed in lepidote trichomes, glandular trichomes (Figure 8d), and laticifers (Figure 8g), which all produced a dark brown to black precipitate after staining with ferric chloride. Stalk cells of lepidote trichomes also appeared to contain phenolic compounds, as these autofluoresced under UV light. Phenolic compounds are common among *Croton* species [22]. These compounds defend the plant against pathogens, parasites, and predators [69]. Furthermore, phenolic compounds from medicinal plants used in traditional medicine are known to possess biological and pharmacological activities [69,70]. The medicinal and cosmeceutical industries have also utilized phenolic compounds, as they are reported to possess antioxidant properties [30]. Lipidic compounds were detected using Sudan III and Nile Blue. Laticifers and stalk cells of lepidote trichomes stained orange with Sudan III, indicating the presence of lipidic components. Lipids were also observed in glandular trichomes (Figure 8e). Nile Blue was used to detect acidic and neutral lipids. Subradial, radial, and central cells of lepidote trichomes and laticifer cells stained blue for acidic lipids. The stalk cells of lepidote trichomes and glandular trichomes (Figure 8c) stained pink, indicating neutral lipids. Others also detected the presence of lipids in these external structures [71]. Vitarelli et al. [44] revealed lipidic compounds in the stalk cells of *C*. *erythroxyloides* using secondary fluorescence. The presence of lipids in the stalk cells of lepidote trichomes is suggested to enforce symplastic transport [44]. Alkaloids, lipids, and phenolic compounds were also detected in the laticifers of *C. echinocarpus* and *C. urucurana* [72]. Sections stained with Ruthenium Red revealed the presence of mucilaginous substances in laticifers indicated by a pinkish red coloration in the cells. Staining with Toluidine blue O resulted in an intensely dark blue/purple coloration of laticifers, indicating that these cells contain macromolecules with free phosphate groups.

### 3.6. Ultrastructure of Lepidote Trichomes

**Figure 9:**
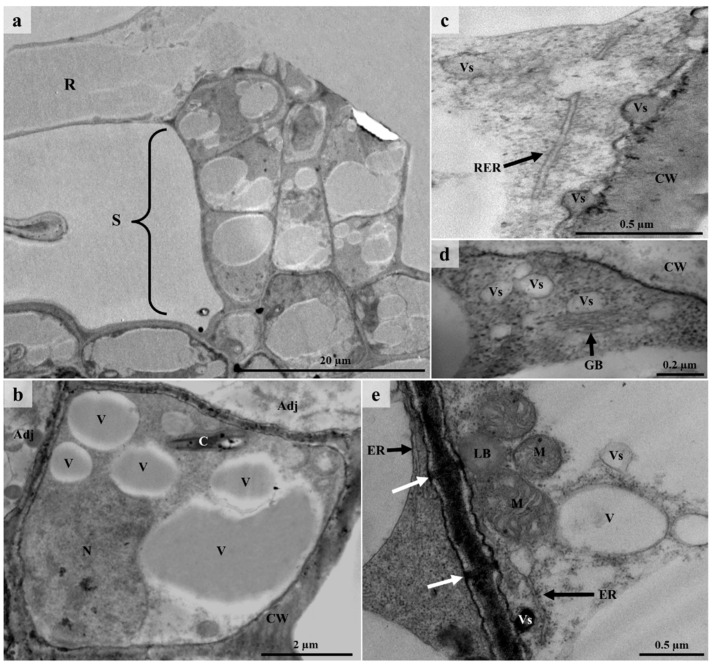
TEM micrographs of lepidote trichome stalk cells. (**a**) Section through the stalk cells and radial cell. Large and small vacuoles surrounded by dense cytoplasm and other organelles can be seen in the stalk cells. (**b**) Single stalk cell containing dense cytoplasm rather numerous vacuoles, a large nucleus, and a chloroplast. (**c**) Rough endoplasmic reticulum and vesicles at the periphery of a stalk cell wall. (**d**) Vesicles and Golgi body present in stalk cells. (**e**) Cell wall between two adjacent stalk cells with visible plasmodesmata (white arrows). Vacuoles, numerous mitochondria, endoplasmic reticulum, and vesicles can be seen at the periphery of these cells. Note the presence of the electron dense vesicle and lipid body next to the cell wall. R—Radial cell, S—Stalk, CW—Cell wall, Vs—Vesicle, V—Vacuole, N—Nucleus, M—Mitochondria, RER—Rough Endoplasmic Reticulum, ER—Endoplasmic Reticulum, C—Chloroplast, GB—Golgi body, LB—Lipid body, Adj—Adjacent cells.

**Figure 10:**
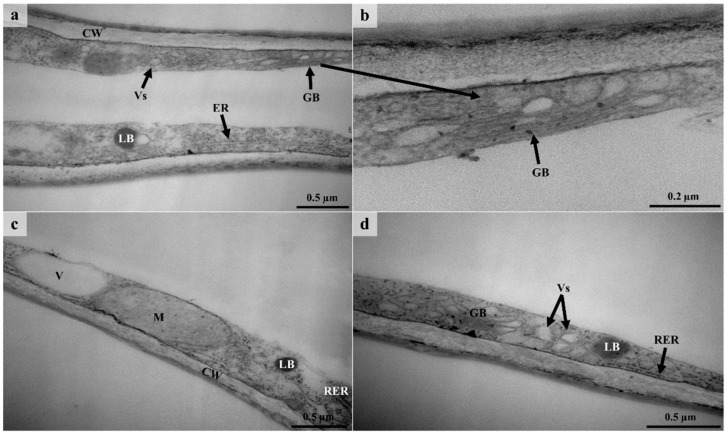
TEM micrographs of lepidote trichome radial cells. (**a**) Radial cell with thickened cell wall containing dense cytoplasm with vesicles, Golgi body, a lipid body, and endoplasmic reticulum at the periphery. The middle of this cell appears to be empty. (**b**) Higher magnification of Golgi body surrounded by dense cytoplasm. (**c**) A vacuole, mitochondrion, lipid body, and rough endoplasmic reticulum present along the radial cell wall. (**d**) Golgi body, rough endoplasmic reticulum, a lipid body, and numerous vesicles along the periphery of a radial cell wall. CW—Cell wall, Vs—Vesicle, V—Vacuole, M—Mitochondrion, RER/ER—Rough Endoplasmic Reticulum/Endoplasmic Reticulum, GB—Golgi body, LB—Lipid body.

TEM revealed the presence of various organelles within the stalk and radial cells of lepidote trichomes (Figure 9 and Figure 10). Stalk cells contained numerous vacuoles which occupied the bulk of the cell (Figure 9a,b). Vacuoles were also present in the radial cells (Figure 10c). Vacuoles play a role in processing secretory material [73,74] (Machado et al., 2005; Huang et al., 2008). Large nuclei with dense nucleoplasm were present in the stalk cells (Figure 9a,b) but were not prominent because of the surrounding dense cytoplasm. Chloroplasts were also observed in the stalk cells (Figure 9b), as they are involved in the production of lipophilic substances [16] (Fahn, 1979). Werker and Fahn [75] (1981) suggested that large amounts of secretory substances may be produced by chloroplasts. Stalk and radial cells contained lipid bodies, several vesicles, rough endoplasmic reticulum, Golgi bodies, and numerous mitochondria (Figure 9c–e and Figure 10). However, within radial cells, the cytoplasm and the various organelles were restricted to the periphery of the cell (Figure 10).

Many of the vesicles in the stalk and radial cells appeared translucent, whilst others contained dense material (Figure 9c–e and Figure 10a,d). These vesicles indicate the secretion of hydrophilic substances and their occurrence close to the plasmalemma suggests granulocrine secretion [15]. The plasmalemma also appeared sinuous, indicating vesicle fusion [76]. Granulocrine elimination of secretions occurs in all secretory cells [16]. Granulocrine secretion is described as the collection of secretory substances in membrane-bound vesicles that either fuse with the plasmalemma or are eliminated by invaginations of the plasmalemma [16]. According to several authors [74,75,77,78], Golgi bodies in secretory trichomes play a role in the production of acidic and neutral polysaccharides. It has been suggested that endoplasmic reticulum is also involved in the production of polysaccharides [75]. The ER produces the protein component of the secretory product which is then transferred to the Golgi body [77]. The Golgi body produces the polysaccharide component which is then transported by the vesicles [77]. Huang et al. [74] suggest that vesicles that are close to the plasmalemma and Golgi body transport the polysaccharide material which is released through granulocrine secretion. Evidence for this is seen in the cells of the lepidote trichomes of *C. gratissimus* (Figure 9d and Figure 10a,d).

Stalk cells contained walls with plasmodesmata (Figure 9e). However, the lateral walls of the stalk cell appeared highly cutinized (Figure 9b). Ascensão and Pais [77] suggested that the presence of plasmodesmata enabled the symplastic transport of precursors. The lignified walls act as an apoplastic barrier to prevent the backflow of secreted substances, as these may be toxic to mesophyll cells [16,42,76]. Although lepidote trichomes are regarded as non-secretory, numerous organelles within the stalk and radial cells (Figure 9 and Figure 10) indicate high metabolic activity [79]. However, much of the activity was in the stalk of the lepidote trichomes. According to Fahn [16], the endoplasmic reticulum and Golgi body are involved in the secretion of hydrophilic substances. On the other hand, various organelles, including the nucleus, mitochondria, Golgi body, endoplasmic reticulum, plastids, and ground cytoplasm, may be responsible for the secretion of lipophilic substances. All these organelles were present in the lepidote trichomes of *C. gratissimus*. Observations from TEM and histochemistry suggest that lepidote trichomes are involved in the synthesis and/or accumulation of secondary metabolites. However, more studies are needed to confirm the secretory mechanism in these trichomes.

## 4. Conclusions

The leaves and stems of *C. gratissimus* possessed lepidote and glandular trichomes, and non-articulated, unbranched laticifers. Lepidote trichomes formed a dense indumentum on the abaxial surface of the leaves which canopied the underlying glandular trichomes. The shield-like structure of lepidote trichomes provides a larger surface area for water absorption and may also function as a protective barrier against external factors and predators. Initially, these structures were thought to be non-secretory, but histochemical and analyses and TEM indicated that lepidote trichomes are metabolically active and produce secondary metabolites which may function as chemical barriers for the leaves as well. Glandular trichomes and laticifers also tested positive for secondary metabolites which possibly contribute to the chemical defense of the plant. The secretory compounds may also possess medicinal properties, which probably explains their extensive use in traditional medicine. However, additional research is required to identify the mode of synthesis of the secretory compounds.

## Figures and Tables

**Table 1:** Histochemical analysis of lepidote trichomes (LT), glandular trichomes (GT), and laticifers (L) of *C. gratissimus* var. *gratissimus*.

Compound Class	Histochemical Test	LT	GT	L	Reaction Observed
Alkaloids	Wagner’s reagent	++	++	++	The stalk of lepidote trichomes and laticifer cells stained orange–brown. Contents of glandular trichomes stained dark brown.
Lipids	Sudan III	+	+	++	Stalk cells and contents of lepidote trichomes stained orange. Lipid droplet in glandular trichome stained bright orange. Laticiferous cells stained orange.
	Nile Blue A	++	++	++	Subradial, radial, and central cells of lepidote trichomes, and laticifer cells stained blue. Stalk cells of lepidote trichomes and contents of glandular trichomes stained pink.
Phenolic compounds	Ferric chloride	++	++	++	Stalk and radial cells of lepidote trichomes stained brown and dark brown, respectively. Contents of glandular trichomes stained dark brown. Laticifers stained dark brown to black.
Lignin	Phloroglucinol	+	NT	NT	Central cells of lepidote trichomes stained light red.
Mucilage and pectin	Ruthenium Red	+	NT	++	The subradial, central, and radial cell walls of lepidote trichomes stained pink. Laticiferous cells stained pinkish red.
Carboxylated polysaccharides, polyuronides, macromolecules with free phosphate groups and polyphenols	Toluidine blue O	++	NT	++	Subradial and central cells of lepidote trichomes stained bright blue. Laticifers stained dark blue.

+ indicates presence of compound class; ++ indicates intense reaction; NT—Not tested.

## Data Availability

The results from this article are a part of MSc thesis “Secretory Structures of *Croton gratissimus* Burch. var. gratissimus (Euphorbiaceae): Micromorphology and Histophytochemistry” by Danesha Naidoo (School of Life Sciences of the College of Agriculture, Engineering and Science, University of KwaZulu-Natal, Westville, South Africa) published online: https://researchspace.ukzn.ac.za/bitstream/handle/10413/17617/Danesha%20Naidoo_2018.pdf?sequence=1&isAllowed=y“ (accessed on 5 February 2023).
